# Feasibility and desirability of scaling up Community–based Health Insurance (CBHI) in rural communities in Uganda: lessons from Kisiizi Hospital CBHI scheme

**DOI:** 10.1186/s12913-020-05525-7

**Published:** 2020-07-17

**Authors:** Alex A. Kakama, Prossy K. Namyalo, Robert K. Basaza

**Affiliations:** 1 Kisiizi Hospital Community-based Health Insurance Scheme, Kisiizi Hospital, Kabale, Uganda; 2 Department of Social Sciences, Ndejje University, Kampala, Uganda; 3 Gudie Incubation Centre, Kira Municipality, Uganda; 4 Uganda Christian University Mukono, Masters of Public Health Leadership Program, Mukono, Uganda

**Keywords:** Community-based health insurance, Universal health coverage, Health financing, Enrolment, Scaling up, Uganda

## Abstract

**Background:**

Community-based Health Insurance (CBHI) schemes have been implemented world over as initial steps for national health insurance schemes. The CBHI concept developed out of a need for financial protection against catastrophic health expenditures to the poor after failure of other health financing mechanisms. CBHI schemes reduce out-of-pocket payments, and improve access to healthcare services in addition to raising additional revenue for the health sector. Kisiizi Hospital CBHI scheme which was incepted in 1996, has 41,500 registered members, organised in 210 community associations known as ‘*Bataka*’ *or ‘Engozi’* societies. Members pay annual premiums and a co-payment fee before service utilisation. This study aimed at exploring the feasibility and desirability of scaling up CBHI in Rubabo County, with specific objectives of: exploring community perceptions and determining acceptability of CBHI, identifying barriers, enablers to scaling up CBHI and documenting lessons regarding CBHI expansion in a rural community.

**Methods:**

Explorative study using qualitative methods of Key informant interviews and Focus Group Discussions (FGDs). Seventeen key informant interviews, three focus group discussions for scheme members and three for non-scheme members were conducted using a topic guide. Data was analysed using thematic approach.

**Results:**

Scaling up Kisiizi Hospital CBHI is desirable because: it conforms to the government social protection agenda, society values, offers a comprehensive benefits package, and is a better healthcare financing alternative for many households. Scaling up Kisiizi Hospital CBHI is largely feasible because of a strong network of community associations, trusted quality healthcare services at Kisiizi Hospital, affordable insurance fees, trusted leadership and management systems. Scheme expansion faces some obstacles that include: long distances and high transport costs to Kisiizi Hospital, low levels of knowledge about health insurance, overlapping financial priorities at household level and inability of some households to pay premiums.

**Conclusions:**

CBHI implementation requires the following considerations: conformity with society values and government priorities, a comprehensive benefits package, trusted quality of healthcare services, affordable fees, trusted leadership and management systems.

## Background

Community-based Health Insurance (CBHI) schemes are famous world over. African countries including Ghana and Rwanda, as well as developed countries like Germany, Japan and China have implemented CBHI schemes as initial steps towards attainment of the national health insurance coverage [1–3]. These CBHI schemes are characterised by common principles such as: risk-sharing, voluntary membership, community solidarity and non-profit [2, 3].

The CBHI concept developed out of a need for financial protection against catastrophic health expenditure of the poor after failure of user fees, tax-based and social health insurance systems [4, 5]. Certainly, CBHI schemes reduce out-of-pocket payments, improve cost–recovery and to some extent influence quality of care [6]. In addition, the schemes improve access to healthcare services and raise additional revenue for the health sector especially in low-income settings [7].

In Uganda, the first CBHI scheme was set up at Kisiizi Hospital and it allowed families to join through community associations or groups of not less than 20 households [8, 9]. Twenty-nine other similar schemes have since been set up in different parts of the country. Membership in these schemes has been staggering at about 155,057 or so people, estimated at about 5–10% of the catchment population in the country [8].

Sustainability and expansion of these schemes has been hampered by numerous operational challenges including: small budgets, rising healthcare costs, small risk pools, irregular contributions, and overutilization of services [10]. The other limitations related to scheme enrolment include: (a) inappropriate benefits package, (b) cultural beliefs, (c) affordability, (d) distance to healthcare facility, (e) lack of adequate legal and policy frameworks to support CBHI, and (f) stringent rules of some CBHI schemes [10, 11]. On the other hand, the registered success has been attributed to: community awareness and understanding of CBHI concept, perceived quality of healthcare services and trust in scheme management [10, 11].

The Kisiizi Hospital CBHI scheme was established in 1996. By the end of 2018, the scheme had 41,500 active members registered through 210 community mutual groups called ‘*Bataka’ or ‘Engozi’* societies in and surrounding areas of Rubabo County [12, 13]. The County has an estimated population of 136,200 people [14]. This puts the estimated total catchment population at only 30%. The scheme was a strategy of reducing the unpaid hospital debts which had risen to about 2.5% of the total annual recurrent hospital costs to less than 1.5% [12]. The scheme members pay annual premiums that range from UGX 11,000 – UGX 17,000 (USD 3–4.7^1^) per person. In addition, the members are required to pay a co-payment fee of UGX 3000 (USD 0.8) per any out-patient visit; UGX 150,000 (USD 41.7) per major surgery including a caesarean section; UGX 10,000 (USD 3.8) per paediatric admission and UGX 30,000 (USD 8.3) per non-surgical adult admission [13].

The abolition of user-fees in all government health facilities has not promoted equitable access to healthcare services in Uganda [15]. The poor and vulnerable still have limited access to healthcare services, and cost is still a key barrier to accessing services [16]. CBHI is being fronted as one of the mechanisms to address these inequities as well as facilitate the introduction of the proposed National Health Insurance Scheme [8].

## Methods

### The aim

To explore the feasibility and desirability of scaling up Kisiizi CBHI Scheme in Rubabo County, Rukungiri District in Uganda. In particular, we explored the perceptions and determined acceptability of CBHI Scheme; identified major barriers and enablers to scaling up Kisiizi CBHI Scheme. We also documented lessons learnt regarding CBHI expansion in rural communities in Rubabo County, Rukungiri District, Uganda.

### Design and setting of the study

In assessing the feasibility and desirability of scaling up of CBHIS, this paper adapted a framework developed by Normand and Weber [17] which applies the considerations highlighted in the decision making phase, in conjunction with the Bowen’s key areas of focus in Figure 1 (Additional file 1). In this paper, the feasibility of scaling up CBHI is defined as how CBHI is feasible in the context of existing constraints; whereas the desirability for scaling up CBHI has been identified in terms of popular support for CBHI, how CBHI fit within health policy objectives and considerations for scaling up CBHI.

We adopted a descriptive research design using mainly a qualitative approach. We explored the possibility and attractiveness of CBHIs in a rural community and this approach allowed for gathering in-depth information since health insurance is fairly a new concept in Uganda. This study was carried out in Rubabo County, Rukungiri District, South Western Uganda. The county forms the primary catchment area of Kisiizi Hospital, a private-not-for–profit facility and other 33 both public and private lower health facilities [13, 14].

### The study population

Included: scheme members and non-members, local and opinion leaders, staff and managers of the scheme, and officials of the District Health Department. Primary participants were selected from three villages with different levels of health insurance coverage, classified as: ‘very low coverage’ (up to 19% insurance coverage), ‘low coverage’ (20–39% insurance coverage) and ‘moderate coverage’ (greater or equal to 40% insurance coverage) [13]. The inclusion of participants from a variety of settings aimed at increasing the validity and credibility of the findings [18]. Participants were selected purposively, using criterion sampling technique. The criterion for selection of Focus Group Discussion (FGD) participants was that one must have lived in the selected village for at least 2 years. Two years were considered an adequate period for a village resident to have reflected on joining or not joining the CBHI scheme. Mixed gender FGDs were conducted; two per village: one for scheme members and another for non-members. Each FGD was composed of six-eight participants. Selection of key informants was based on leadership positions in the target office or in the community and their availability and willingness to be interviewed. Secondary participants were interviewed through face-to-face key informant interviews (KI) using a key informant guide. These included officials from the District Health Department, community opinion leaders, staff and managers of the scheme. The key issues included in both key informant and FGDs guide were topics in Figure 1.

### Data collection, management, analysis and quality control

Seventeen (17) key informant interviews and six focus group discussions were conducted by of the investigators and a Research Assistant using a topic guide (Additional file 2). The Assistant was an experienced researcher in qualitative studies in health financing. Each FGD had six to eight participants (*n* = 47) and discussions were held at community centres. The key informant interviews were held at the offices of the respective participants. The discussions were moderated by the research assistant who introduced himself and the goal of the project before the discussion. The discussions were audio recorded and took 1–2 h. The whole process of data collection took place from 11th December 2018 to 20th March, 2019. The investigators used a grounded theory in coming up with research tools. The theory further helped the investigators in exploring participants’ perceptions and understanding of the scheme processes, motivations and systems. This study focussed on appropriate key areas for conducting feasibility studies, especially: acceptability, practicality and expansion [19].

Data analysis was based on the individual or group participant’s opinions and perceptions of the scheme. This is followed the process of transcribing and checking, open coding, identification of patterns/themes and finally summarising the data [20]. The audio-recorded data was converted into written text to facilitate analysis. Coding was done manually. The investigators read through the transcripts at least three times so as to make meaning of the entire story before generating themes. The investigators made memos in the margins which helped to identify common words, phrases and the differences which helped to generate common themes, patterns within the responses. The generated themes were then linked to the study objectives. The investigators chose to present empirical data with both verbatim quotes from individual participants as well as with excerpts of the discussions between the participants. This was aimed at making the results more valid reflection of the empirical data. The investigators decided to mark the excerpts from the discussions ‘member FGDs’ or ‘non-member FGDs’ to represent focus group discussions for health insurance members or non–insurance members respectively. In addition, for reporting purposes, the authors have maintained the anonymity of the participants.

The quality control involved pre-testing in Rugarama sub-County, Ntungamo District. This area was chosen because; (a) it is close to Kisiizi hospital and falls outside the study area, (b) It has both scheme members and non-scheme members. The tools were then edited to remove errors, ambiguity, repetitions and made respondent friendly. An orientation training in data collection techniques was conducted for the research assistant. During the interviews, first the strategy of member checking was applied, where the investigators would recite back the responses in order to seek participant’s clarification before the end of the interview. The second strategy was to listen again to the audios after the first draft of the script was written to ensure that whatever each participant said was fully captured. Thirdly, the investigators adopted a strategy of bracketing, where the researcher’s experiences, beliefs, values and feelings were deliberately put aside in order to accurately describe participants’ opinions and perceptions. Triangulation was obtained through separate focus group discussions with scheme and non-scheme members so as to compare their views. Also use of KII and FGDs provided for triangulation.

## Results

### Background characteristics of participants

A total of 64 participants took part in this study and their characteristics are presented in Table 1. Up to 26.6% (17) were key informants and 73.4% (47) participated in the focus group discussions. Up to 29.7% (19) were males and 70.3% (45) were females. Key informants’ age ranged from 27 to 69 years (mean = 48, SD = 10.5).

**Table 1:** Participants’ background characteristics

	No.	%
**Gender**		
Male	19	29.7
Female	45	70.3
**Type of participant**		
KI	17	26.6
FGDs	47	73.4
**Employment status of participants**		
Formal employment	10	15.6
Informal employment	54	84.4
**Educational level of key informants**		
Primary level (≤7 years of education)	3	17.7
Secondary level (> 7–13 years of education)	4	23.5
Tertiary/University level (> 13 years of education)	10	58.8
**Occupation of key informants**		
Peasant farmers	8	47.1
Vendors	3	17.6
Health managers	6	35.3
**Age of key informants (years)**		
20–29	2	11.8
30–39	4	23.5
40–49	6	35.3
50 and above	5	29.4
**FGDs participants**		
Scheme members	22	46.8
Non-Scheme members	25	53.2
**Health insurance coverage in the villages**		
Moderate (≥40%)	18	38.3
Low (20–39%)	16	34.0
Very low (< 20%)	13	27.7

Key informants included three (3) officials in the District Health Department, (3) managers and staff of Kisiizi Hospital CBHI scheme and eleven (11) community leaders. Up to 46.8% (22) of the participants in the FGDs were members of the Kisiizi Hospital Health Insurance Scheme for at least 2 years and 53.2% (25) were non-scheme members. Up to 38.3% (18) were from a village with moderate whereas 34.0% (16) and 27.7% (13) were from villages with low and very low health insurance coverage.

### Assessing desirability of scaling up the Kisiizi Hospital CBHI scheme

#### Community acceptability and support to the Kisiizi Hospital CBHI scheme

Participants expressed support for the scheme in line with the scheme’s objective of extending financial support to the members to fund hospital care. For instance, member FGD 1 mentioned that: “the scheme helps us to clear bigger part of the hospital bill when our mothers have caesarean sections”. The Group further added: “the scheme has been paying for us hospital fees for all patients including those that require surgery”. On the same note, some opinion leaders stated that: “we have seen the scheme helping some people who would not have managed to pay hospital bills especially for complicated healthcare services” (KI 5). “Most of the people in this community no longer sell family land or borrow from dubious money lenders to finance hospital care” (KI 8). In a similar way, key informant 3 mentioned that: “the community members understand that the scheme is here to help them”.

It was stated in non-member FGD 1 that: “we hear that members get free services at Kisiizi Hospital”. Relatedly, participants in non-member FGD 3 mentioned that: “we are told that scheme members get discounts and pay little amounts of money for services but we are not sure of how this works”. Thus both members and non-members have positive attributes to acceptability of the scheme.

#### Conformity with national health policies and guidelines

Key informants stated that the scheme’s objective of promoting access to quality healthcare services at affordable cost, is in line with local and national government priority of promoting universal health coverage. For instance, key informants 2, 3, and 5 mentioned that: “the government priority is to promote universal health coverage. The people should be able to access quality healthcare service at affordable cost”. In addition, key informant 7 stated that: “The CBHI scheme provides an opportunity to all people especially the poor to get quality services at Kisiizi Hospital, at a very affordable cost”. Another key informant 8 revealed that: “as leaders we are happy that our people are able to get good services at a cost they are able to meet”.

#### Conformity with society values and culture

Participants uniformly stated that the scheme’s ideology and methods of work are very similar to the methods, practices and objectives of the local community associations which offer financial and material support to grieving families in times of death of loved ones or even in times of illness. Key informant 11 stated that: “the scheme works more like our *‘engozi’* groups, where we support each other with finances and food items during funerals and sickness”. In addition, during the FGD, members emphasised that: “the scheme operates more like our ‘*Bataka/engozi’* association; we pool funds and food items to help members during funerals of loved ones (member FGD 2)”.

#### Acceptability of the benefits package

The scheme members agreed that the benefits package meets the healthcare needs of most of the people in the community. They further stated that the scheme offers insurance cover for common acute illness, accidents/ trauma and maternity services with some exclusions of non-communicable diseases like high blood pressure and diabetes. One FGD participants stated that: “the scheme pays for common services except high blood pressure and diabetes” (member FGD 2). Relatedly, key informant 4 said that: “the scheme covers out-patient services, deliveries and emergency services”. However, participants were interested in expansion of the benefit package. This was seen in some instances where they requested for coverage of non-communicable diseases through giving such patients a subsidy. In member FGD 2, participants intimated that: “members with hypertension and diabetes should also be given a significant subsidy”. On the other hand, non-members had mixed ideas regarding the benefits package. For instance, FGD 3 was not sure of the benefits package: “we hear that the scheme covers simple illness like malaria but not bigger conditions like hypertension and diabetes which are common in our community”. They were not sure of what should be done when member pays a premium and does not fall sick. … “what happens to the money when I do not fall sick? it is never refunded” (non-member FGD3).

#### Coping mechanism for non-members of the scheme

Concerning households’ mechanisms to mobilise funds for healthcare, it was mentioned that most non-member families either borrow or sell family property especially land. For instance, in non-member FGD 2, it was mentioned that: “it is difficult to raise adequate funds to pay off hospital bills without borrowing or selling family property”. A non-member FGD1 opined that: “Some people sell goats or cows to pay hospital bills”. Similarly, another non-member FGD 3 mentioned that: “some of us have had to sell part of our banana plantations to settle hospital bills when our wives or daughter in-laws go for delivery”. Further still, key informant 9 also stated that: “most people borrow funds from money lenders at high interest rates and use land as a collateral to settle healthcare bills”.

### Assessing feasibility of scaling up the Kisiizi Hospital CBHI scheme

This section presents findings on the practicability of CBHI implementation amidst existing constraints. Feasibility considerations were categorised as either enablers or barriers to this scheme.

#### Enablers to scaling up Kisiizi Hospital CBHI scheme in local communities

##### Existing community associations or groups

Participants mentioned that the scheme worked with the existing *‘Engozi’* groups to promote the health insurance agenda and enrol members. Key informant 4 had this to say: “there is at least one community association in each village in Rubabo”. “It was easy to penetrate the community through the existing *‘Engozi’* groups, which had to add health insurance into their development agenda” (KI4). Participants in member FGD 2 mentioned that: “almost every household in this community belongs to a certain *‘engozi’* group; indeed, Kisiizi scheme worked with these groups to enrol us”.

##### Trusted quality of services at Kisiizi Hospital

It was found that participants trust the quality of services provided at Kisiizi Hospital. Most participants mentioned that Kisiizi Hospital offers good quality services. During member FGD 1, participants mentioned that: “Kisiizi Hospital offers the best healthcare services in and around Kigezi region”. Key informant 2 added that: “we trust the services at Kisiizi Hospital”. Further still, key informant 9 stated that: “the doctors and nurses at Kisiizi Hospital are always available”. In the same way, non-member FGD 3, mentioned that: “Kisiizi Hospital has good doctors and machines. Most people get healed from Kisiizi Hospital”. According to key informant 2: “trust in the quality of services offered by Kisiizi Hospital has been a key factor to the success of the scheme”.

##### Affordable premiums

Most participants stated that the premiums are largely affordable. For instance, member FGD 3 mentioned that: “the premiums are affordable to many families in this village”. In a related way, key informant 9 said: “the premiums are not that high; most people can afford”. It was also found out that scheme members were actively involved in setting the premiums. Key informant 3 mentioned that: “all insurance payments are set by the executive committee in collaboration with hospital management, but approved by the annual general assembly of members, with an agreement that the set fees are affordable to majority of the households in our catchment area”. In a similar way, key informant 3 and 13 stated that: “premiums are purposely kept in affordable ranges so that the majority of the households can enrol”. On the other hand, non-members mentioned that: “the premiums should be further reduced to UGX 5,000 (USD 1.3) per person per year” (non-member FGD 1). Relatedly, it was mentioned in another non-member FGD 3 that: “fees are fairly affordable for small families but not for large families”.

##### Strong governance and management structures

It was established that the scheme is governed by members through an elected executive committee of 11 members. The main responsibilities of the executive committee include: making policies, evaluating proposals for insurance fees reviews, auditing scheme finances, and providing regular feedback about the services. This makes them have a sense of ownership of the scheme. “The scheme belongs to the members and Kisiizi Hospital helps to administer it” (KI 1, 16,17). In addition, key informant 7, mentioned that: “The hospital management consults with the executive committee in case of need to review fees. Secondly, all fees changes are presented to the members in the annual general meeting for approval”.

#### Barriers to scaling up the Kisiizi Hospital CBHI scheme in local communities

##### Long distance and high transport costs to Kisiizi Hospital

Kisiizi Hospital is located over 30 Km away from the main road and over 50 Km from the urban centre. Participants mentioned that it is difficult to travel to and from Kisiizi Hospital due to unreliable public transport means. “It is difficult to travel to and from Kisiizi in the afternoon and night hours” (member FGD 2). In non-member FGD 3, it was mentioned that: “public transport costs to Kisiizi Hospital for a patient and one care taker are higher than costs of medical care in a nearby clinic”. The group added that “it is relatively costlier to go to Kisiizi Hospital than paying for services at a private clinic”.

##### Low levels of knowledge, negative attitude and beliefs about health insurance

Some participants questioned the motive of pooling funds annually and expressed fears of the accountability of these funds. For instance, non-member FGD 1, asked: “where does the money go if one does not get sick throughout the year?” In a similar way, non-member FGD 2 mentioned that: “why should I pay before I get sick?” In addition, a few participants who were non-members claimed to have witnessed segregation of insurance patients from cash –paying patients at Kisiizi Hospital. For instance, a non-member FGD 1 mentioned that: “the health workers at Kisiizi Hospital offer better services to patients who pay cash than those in health insurance”. They added that: “scheme members are given only panadol which costs less”.

##### Inability to pay premium

Participants stated that a few families in the local communities have failed to enrol into the scheme due to failure to raise premiums. Key informant 6 mentioned that: “the poorest families especially those that do not belong to community associations cannot afford to pay premiums”. Secondly, overlapping of school fees periods with membership renewal period was mentioned as another factor for failure to renew scheme membership. Key informant 3, 5, 7 and 11, mentioned that: “some families have dropped out of the scheme due to failure to pay premium, especially during periods when children are returning to school and parents have to pay school fees”.

## Discussion

### Desirability of scaling up the Kisiizi Hospital CBHI scheme

Participants revealed acceptability and support for the scheme, linked to the scheme’s ability to offer financial support to clear hospital bills. This research finding is in agreement with a study done in Ghana that indicated that people enrolled were happy with the scheme because the scheme offered financial protection of their families by paying for their healthcare bills [21]. Secondly, the findings show that the scheme objectives and processes contribute towards attainment of the government agenda of promoting universal access to healthcare services at low cost. The findings of this study are supported by national policies and strategic plans. The Country long term plan, the Uganda Vision 2040, identifies health insurance as one of the key strategies for alleviating high costs on health care by households and enhancing access to affordable health services for all [22]. Thirdly, the respondents pointed out that hospital CBHI Scheme ideology and methods of work are in line with the practices and objectives of the local community associations, *‘Engozi groups’,* which are part of the society norms*.* The findings of this study agree with previous research that indicated that community social capital enables better access to care [23, 24].

Concerning the benefits package, respondents intimated that the scheme offers cover for common illnesses and health conditions. The findings of this study agree with previous studies that people in rural areas preferred a benefit package which is comprehensive in nature, offering inpatient, outpatient and emergencies services [25].

Regarding the available health financing alternatives, hospital level services can only be easily accessed at Kisiizi Hospital which charges user fees. The research findings agree with another study that showed that 53% of the patients getting surgery in Ugandan hospitals borrow money to finance their care and 21% sell family property [26]. Additionally, the findings are also in agreement with a study by Natasha which indicated that user fees deterred utilisation of services while prepayment or insurance schemes offered potential for improving access [27].

### Feasibility of scaling up the Kisiizi Hospital CBHI scheme

The scheme operates through local associations to promote the health insurance agenda and enrol members. This was considered as key factor in the success of this scheme. The findings are in agreement with other studies which emphasise that CBHI schemes can be built on existing social capital to increase coverage by enrolling households through community associations [23]. Secondly, it was revealed that most people were comfortable with the level of quality of services at Kisiizi Hospital. Previous studies indicate that perceptions, attitudes and beliefs about service providers strongly influence the households’ decisions to enrol and remain enrolled into the scheme [21, 25]. Thirdly, participants revealed that insurance fees are affordable to many households in the local community. This was because fees were set by members and administrators of the scheme basing on affordability as a key factor. Existing literature indicates that ability to pay premium and co-payment fees is a strong enabling factor to scheme enrolment [9]. In addition, results indicated that members are actively involved in the management of the scheme through an elected executive committee. Previous studies have indicated that: trust in the management of the scheme especially the integrity of the scheme managers strongly influences a household’s decision to enrol into the scheme [28], and having robust management or administrative structures is essential to scheme implementation and influences sustainability of the CBHI schemes [10].

However, the expansion of the Kisiizi Hospital CBHI Scheme continues to face a number of deterrents. The high transport costs to and from the hospital masks the visible advantages of scheme membership. This finding is in line with existing literature that large distance to in-network health facilities constitute a significant obstacle to enrolment, and even a reason for non-renewal of membership into CBHI schemes [10]. Secondly, some respondents were not very convinced of the reasons for pooling funds in preparation for unforeseen illness, and the subsequent accountability of the funds at the end of a financial year. These statements depict lack of knowledge on the concept of health insurance, especially among non-insured community members. This was also pointed out in a related study in Ghana where low levels of knowledge led to doubt and influenced negative attitudes towards enrolment into CBHI schemes [21], while consumer awareness and understanding of the concept of health insurance are significant determinants of scheme uptake [10]. Lastly, it was revealed that some families are not able to raise premium and have failed to enrol into the scheme and yet others drop out in the periods where there are pressing issues like when paying school fees for children at the beginning of the term. This tallies with the findings of a study of CBHI schemes in sub-Saharan Africa that underscored families dropping out of CBHI schemes due to difficulties to meet subscription fees [29], and the policy to pay annual premiums in one payment is a significant obstacle for some families [10].

### Lessons learnt

Amidst free healthcare in government facilities, it was learnt that CBHIs have continued to survive for a number of years. This is because people are willing to pay as long as they receive the required quality services. This finding is in agreement with a study done in Ghana which observed that households were willing to pay the required premiums on condition that the required quality services were delivered [21]. Another study done in Nigeria was in agreement with this finding as well [30]. Thus, policy makers need to think ways of exploring how to charge those already in CBHI schemes, and plan how they could be integrated into the proposed National Health Insurance Scheme but ensure that they satisfy members with good quality services. Secondly, it was learnt that the active involvement of the scheme members in the management of the scheme improved trust and therefore contributed to the continuity of the scheme. Their involvement improves on flow of information from the scheme to community members, accountability and transparency among others. It is partly the reasons why Kisiizi CBHI has continued to exist for the past 24 years. This finding is in line with a study done in Tanzania [31] which indicated that district managers were not trusted by the local people and as such it affected enrolment and general performance of the scheme. Policy makers need to explore ways of integrating community representation into the proposed National Health Insurance Scheme if people with low levels of income are to accept the scheme. Thirdly, even though Kisiizi CBHI has been in existence for the past 24 years, there are people who still don’t appreciate and understand the need for pooling healthcare risks. It was noted that even some members have no full understanding of health insurance principles. This implies that health insurance education should be a continuous process throughout the life of any scheme. This is in line with a number of previous studies that indicated that there may be lack of understanding and limited knowledge on health insurance principles among the insured and the non-insured [21, 29, 32]. Subsidization of the poorest of the poor came up as an issue. Also, it was learnt that since CBHI involve a payment of premium, there are those who will never join: the poorest segments of the population or say those with low levels of income. This is because they can never afford to pay however small the premium may be. This lesson is in agreement with the findings in a study that assessed the ways the poor can be integrated into health insurance in Africa [33].

### Strengths and limitations of this study

The strength of this study lies in its methodology and includes: involving participants from different communities with different insurance coverage. Included in the study were insured and non-insured respondents and thus considered comparative experiences of both groups. This enriched the study findings on the feasibility and desirability of scaling up the CBHI scheme. In addition, triangulation provided an opportunity to compare the study findings.

This study had limitations: The young female respondents could not express themselves openly in presence of their mother-in-laws and father-in-laws during FGDs. The research team had to explain to the entire group that the views presented during the discussions were group views and not attributed to an individual and matters of health affect both the elderly and young equally. The study was qualitative and could have been enriched by quantitative inputs. This research work did not receive any external funding. Therefore, the research team did not have adequate funds for the study which could have been used for deeper exploration of cultural aspects of the population that affect the desirability of scaling up the scheme among other attributes [10, 11]. However, the investigators exercised strict budget control on the available funds.

## Conclusion

The study findings have implications to the scheme promoters, policy makers, scholars on the on the topic of desirability and feasibility of CBHI scheme in similar settings and provides insights of key issues to consider. The enablers have been that CBHI came in handy to fill a health financing gap in communities hitherto with limited free healthcare. Importantly, the scheme’s objective of promoting access to quality healthcare services at low cost is in line with local and national government priority of promoting universal health coverage. The scheme’s ideology and methods of work are very similar to the methods, practices and objectives of the local community associations. Indeed, there are still barriers to scaling up of CBHI; that is why only 30% of the targeted population is enrolled into the scheme.

Arising from this study, the authors recommend that the scheme promoters could explore how to link the current hospital services provided by the scheme to first line health services (health centres, general practices, clinics and the like) so as to address the current huddle of distance and related costs. Such an intervention could strengthen referral and counter referral services and possibly contribute to improvement of members’ satisfaction. Indeed, further research by the scheme promoters, policy makers and scholars could address how to expand the scheme to cover the whole district. In the same vein, alternative benefit package could be explored or other ways of providing insurance cover for the chronic diseases mentioned. Members with such diseases are relatively the most in need of health insurance because the costs of care of chronic diseases which are largely non-communicable ones is higher than most communicable diseases.

## Supplementary information

**Additional file 1: Figure 1.** Issues considered when accessing desirability and feasibility of scaling up Kisiizi CBHI Scheme.

**Additional file 2.** Consent for and Topic guide for a study on the Feasibility and Desirability of Scaling up Community –based Health Insurance (CBHI) in rural communities in Uganda; Lessons from Kisiizi hospital CBHI scheme.

## Data Availability

The topic guided is provided as additional file 2. The datasets used and analysed during the study are available from the corresponding author on reasonable request.

## Abbreviations

CBHI: Community based Health Insurance
FGDs: Focus group discussions
KI: Key informant
UBOS: Uganda Bureau of Statistics
UGX: Ugandan Shillings
USD: United States Dollars

## Footnotes

1 1USD = UGX 3800
