# Supplementary material for: Feasibility and desirability of scaling up Community–based Health Insurance (CBHI) in rural communities in Uganda: lessons from Kisiizi Hospital CBHI scheme
Source: BMC Health Serv Res. 2020 Jul 17;20:662. doi: 10.1186/s12913-020-05525-7 (PMC7367343; doi:10.1186/s12913-020-05525-7)
Supplement: Supplementary file 1 — Additional file 1: Figure 1. Issues considered when accessing desirability and feasibility of scaling up Kisiizi CBHI Scheme. [file 12913_2020_5525_MOESM1_ESM.docx]

**Additional file 1**

**Decision Phase**

- Assessing desirability for scaling up CBHI
  - - *Is there popular support for CBHI?*
      - Level of awareness and understanding
      - Presence of feasible financing alternatives?
    - *Does CBHI fit with health policy objectives*
    - *Considerations for scaling up CBHI*
- Acceptability of the benefits package
- Stake holder’s interests
- History, culture and societal values
- Assessing feasibility of scaling up CBHI
  - - *Is CBHI feasible in the context of existing constraints*
- Administrative and management capacities
- Service delivery at peripheral levels
- Contributions and insurance fees
- Level of awareness and understanding
- Trust in the quality of service provider

## **Figure 1. Issues considered when accessing desirability and feasibility of scaling up Kisiizi CBHI Scheme** (adapted from Normand & Weber, 1994), on reference list as [17].
