# Supplementary material for: Feasibility and desirability of scaling up Community–based Health Insurance (CBHI) in rural communities in Uganda: lessons from Kisiizi Hospital CBHI scheme
Source: BMC Health Serv Res. 2020 Jul 17;20:662. doi: 10.1186/s12913-020-05525-7 (PMC7367343; doi:10.1186/s12913-020-05525-7)
Supplement: Supplementary file 2 — Additional file 2. Consent for and Topic guide for a study on the Feasibility and Desirability of Scaling up Community –based Health Insurance (CBHI) in rural communities in Uganda; Lessons from Kisiizi hospital CBHI scheme. [file 12913_2020_5525_MOESM2_ESM.docx]

# Consent for and Topic guide for a study on the Feasibility and Desirability of Scaling up Community –based Health Insurance (CBHI) in rural communities in Uganda; Lessons from Kisiizi hospital CBHI scheme

**Informed Consent Form**

Hello. My name is Alex Kakama. I would like to ask you a few questions related to this study. The aim of this study is to explore whether scaling up CBHI of Kisiizi Hospital in Rubabo County is feasible and desirable.

**Procedures:**

I will ask you some questions that relate to community health insurance of Kisiizi hospital. Your genuine responses will be greatly appreciated. Your participation is absolutely voluntary and there is no penalty for refusing to take part.

**Confidentiality:**

If you accept to participate in the interview, I will ask you questions privately without the presence of anyone else and in a place where our discussion cannot be overheard. All information that I record will be kept confidential. Your name will not be used and you will not be identified in any way.

**Risks/discomfort**

There is no serious risk to you if you agree to participate in this interview activity. Some of the questions may be sensitive for instance, your experience with health care services at Kisiizi Hospital. I want to assure you that all information collected will be kept confidential.

**Benefits of this study**

This study may help policy makers in coming up alternative ways of financing health care services in Uganda.

May I proceed with the interview?

Yes (participant signs) --------------------------------

NO ---------------------------------------------- (end interview).

***The participant’s signature above verifies that informed consent has been obtained***

#

# Key informant interview guide

# Feasibility and Desirability of Scaling up Community –based Health Insurance (CBHI) in rural communities in Uganda. Lessons from Kisiizi hospital CBHI scheme.

**Section A: Desirability of CBHI**

1. Can Community Based Health Insurance scheme attract popular support or be acceptability in this community?

**Probe for:**

1. *What are the attitudes of community members about Health Insurance?*
2. *How does it fit in the society values and culture?*
3. *How does CBHI fit with health policies in Uganda?*
4. *How do households in this community fund their healthcare services?*
5. *What do you think are the interests of the following stake holders?*
6. *Views of political leaders in this community in relation to healthcare services*
7. *Views of Community members*

**Section B: Feasibility of CBHI**

1. Is it possible to establish and sustain a Community Based Health Insurance Scheme in this community?

**Probe for:**

1. *What do you think would hinder establishment of health insurance in this community?*
2. *What factors would promote establishment of health insurance in this community.?*
3. *What is the people’s level of awareness about health Insurance?*
4. *What percentage of families in this community would be able to pay for health insurance?*
5. *Where do people in this community go for healthcare services?*
6. *What would people want to have in the benefits package of Health insurance?*

# Focus Group Discussion Guide

# Feasibility and Desirability of Scaling up Community –based Health Insurance (CBHI) in rural communities in Uganda. Lessons from Kisiizi hospital CBHI scheme

1. Barriers and enablers to Scaling up CBHI in this community.

Probe:

1. *What do you think would hinder establishment of health insurance in this community?*
2. *What factors would promote establishment of health insurance in this community?*
3. How are the families in this community organized to help each other during illness or difficult times?
4. What do people in this community say about the idea of pooling money for health insurance?

Probe: *What are the attitudes of community members about Health Insurance?*

1. What is your opinion about the benefits package in CBHI of Kisiizi hospital in relation to value for money?

Probe: *What would people want to have in the benefits package of Health insurance?*

1. What is the opinion of people on quality of services at Kisiizi Hospital?
2. Where do people in this community go for healthcare services
3. What is the peoples of awareness about health Insurance?
